# Statistical Modeling of Heart Rate Variability to Unravel the Factors Affecting Autonomic Regulation in Preterm Infants

**DOI:** 10.1038/s41598-019-44209-z

**Published:** 2019-05-22

**Authors:** Rohan Joshi, Deedee Kommers, Chengcheng Guo, Jan-Willem Bikker, Loe Feijs, Carola van Pul, Peter Andriessen

**Affiliations:** 1 Department of Industrial Design, Eindhoven University of Technology, Eindhoven, The Netherlands; 2 Department of Clinical Physics, Máxima Medical Centre Veldhoven, Veldhoven, The Netherlands; 3 Department of Family Care Solutions, Philips Research, Eindhoven, The Netherlands; 4 Department of Neonatology, Máxima Medical Centre, Veldhoven, The Netherlands; 5 Department of Applied Physics, Eindhoven University of Technology, Eindhoven, The Netherlands; 6 Department of Electrical Engineering, Eindhoven University of Technology, Eindhoven, The Netherlands; 7 Consultants in Quantitative Methods, CQM BV, Eindhoven, Netherlands

**Keywords:** Predictive markers, Biomedical engineering

## Abstract

Analyzing heart rate variability (HRV) in preterm infants can help track maturational changes and subclinical signatures of disease. We conducted an observational study to characterize the effect of demographic and cardiorespiratory factors on three features of HRV using a linear mixed-effects model. HRV-features were tailored to capture the unique physiology of preterm infants, including the contribution of transient pathophysiological heart rate (HR) decelerations. Infants were analyzed during stable periods in the incubator and subsequent sessions of Kangaroo care (KC) – an intervention that increases comfort. In total, 957 periods in the incubator and during KC were analyzed from 66 preterm infants. Our primary finding was that gestational age (GA) and postmenstrual age (PMA) have the largest influence on HRV while the HR and breathing rate have a considerably smaller effect. Birth weight and gender do not affect HRV. We identified that with increasing GA and PMA, overall HRV decreased and increased respectively. Potentially these differences can be attributed to distinct trajectories of intra- and extrauterine development. With increasing GA, the propensity towards severe HR decelerations decreases, thereby reducing overall variability, while with increasing PMA, the ratio of decelerations and accelerations approaches unity, increasing overall HRV.

## Introduction

Continuously monitoring heart rate variability (HRV) offers an opportunity to non-invasively track the regulatory activity of the autonomic nervous system. For many decades, the visual and mathematical analysis of HRV has been employed across diverse clinical specialties such as obstetrics, cardiology and intensive care^1^. Furthermore, HRV has the diagnostic and prognostic potential for detecting and monitoring dysregulation due to disease across a broad spectrum of the population including fetuses, neonates and adults^1,2^. For instance, myocardial infarction in adults is associated with reduced HRV which is predictive of mortality^3,4^.

With regard to infants admitted to neonatal intensive care units (NICUs), estimates of HRV are known to be predictive of sepsis, with clinical studies showcasing that continuously displaying HRV-related scores can reduce sepsis-associated mortality^5^. Since HRV is sensitive to dysregulation arising due to sepsis, characteristic changes in HRV may precede the manifestation of clinical signatures of the disease and can thus provide clinicians with a window of opportunity for preemptive therapeutic intervention. Notably, though, conventional features of HRV, originally developed for adults, may not be readily interpretable in NICU patients, because the vast majority of NICU patients are born prematurely (gestational age <37 weeks), and the characteristics of HRV in preterm infants are different from those in adults^6–8^. Primarily, this is because the autonomic nervous system of preterm infants is underdeveloped and the multiple feedback and control loops responsible for homeostasis are not yet fully developed and may not work synergistically^9,10^.

Concerning HRV, a commonly used feature – the standard deviation of normal-to-normal (SDNN) RR intervals, where R is the peak of a QRS complex of the electrocardiogram (ECG), characteristically reflects overall HRV and is lowered in patients with myocardial infarction^11^. However, in septic infants, reduced HRV, even though obvious to the eye, may not be fully captured by SDNN^12^. This is because, unlike healthy adults, who exhibit a heart rate (HR) pattern with an equal number of decelerations and accelerations, preterm infants are susceptible to transient pathophysiological decelerations in HR^13^. Such decelerations potentially result in substantially large values of SDNN, even in the absence of the small increases and decreases in HR that characterizes healthy HRV, and which SDNN was initially designed to capture^2^. Likewise, while SDNN is usually lowered in stressed adults^14^, SDNN in preterm infants reduces during comfort, as evidenced by studies analyzing Kangaroo care (KC)^6,8^ – a routinely employed therapeutic intervention, known to enhance comfort and improve autonomic regulation – during which diaper-clad infants are held prone on the bare chest of one’s parent (typically) for extended periods^15,16^.

Further adding to the complexity of interpreting HRV in preterm infants is the effect of demographic factors such as gender and maturation-related variables like gestational age (GA), postmenstrual age (PMA) and birth weight (BW) that significantly modulate HRV even in term-born infants^9,17–19^. Additionally, preterm infants exhibit a broad range of HR and breathing rate (BR), the influence of which on HRV remains unclear^20^. A better understanding and quantification of the effect of these cardiorespiratory regulatory mechanisms can help improve caregiving by enabling the tracking of maturational changes as well as subclinical signatures of disease.

In this study, we aim to characterize the influence of several demographic (GA, PMA, BW, gender) and cardiorespiratory factors (HR and BR) on the autonomic regulatory mechanisms of preterm infants, as measured by features of HRV. We develop a statistical model to describe the effects of these factors on several features of HRV, including features specifically designed to capture the contribution of transient HR decelerations. Further, we stratify the analysis for periods when the infant was in the incubator, which is where an infant remains for the majority of the day versus periods of KC – a period of enhanced comfort.

## Materials and Methods

### Study design

**Figure 1:**
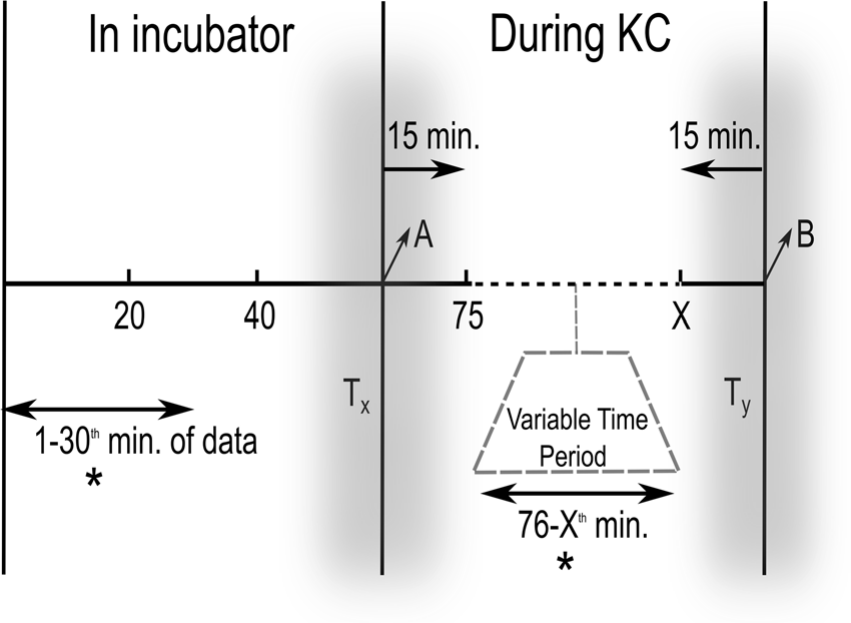
An illustration of the methodology. HRV during periods in the incubator were modeled using the first 30 minutes (indicated with an asterisk) of data from the period in the incubator during which, by design, the infant was not being handled, thus yielding normative values of HRV. The first and last 15 minutes of data during KC were excluded; the rest of the period was also used for statistical modeling (indicated with an asterisk). The duration of KC could vary from one session to the other. Tx and Ty represent the periods of transfer from the incubator to the parental chest and vice versa. ‘A’ refers to the first minute on the parent’s chest; ‘B’, to the first minute back in the incubator.

To investigate autonomic regulation in preterm infants, we conducted a large observational study in the level III NICU of Máxima Medical Center, the Netherlands. We analyzed HRV in two contrasting conditions – in the incubator versus during KC. Routine patient monitoring (IntelliVue MX 800; Philips, Böblingen, Germany), including the acquisition of the ECG waveforms (sampling frequency of 250 Hz), the HR, and the BR (processed via the patient monitor at a resolution of 1 Hz) continued throughout the admission of the infant, including during KC. All data were saved in a data warehouse (PIIC iX, Data Warehouse Connect, Philips Medical Systems, Andover, MA) from which it could be retrospectively acquired. For one year (between Aug 2017 to Aug 2018), parents were requested to annotate the precise start and end times of KC (placement on the parental chest and back in the incubator respectively) in a bedside diary. For all fully annotated KC sessions, patient monitor data, beginning from the one-hour period before the start of KC (henceforth referred to as the pre-KC period) until up to one hour after KC were extracted for analysis. As detailed in previous studies, in our unit, as a matter of protocol, nursing care (typically scheduled for once every two hours) takes place immediately before KC^6,21^. Therefore, the first 30 minutes of the pre-KC period is typically free of nurse handling (a stressful experience)^22^ and is considered to be an epoch *representative* of the incubator environment^6,8^. The periods of transfer, to and fro from the parental chest, can disrupt regulation and are likely stressful^6^ – therefore we consider the entire period of KC, excluding the first and last 15 minutes, as *representative* of the KC-period. This study design is schematically shown in Fig. 1.

### Patient population

All infants admitted to the NICU during the study period were considered eligible for enrolment into the study. Therefore, all parents were requested to participate by annotating KC sessions. Certain KC-sessions were retrospectively excluded such as those performed during serious clinical conditions (e.g., sepsis, necrotizing enterocolitis, intraventricular hemorrhage grade III/IV) or conditions that interfered with breathing (mechanical ventilation). Similar to previous research, we also excluded KC sessions that were shorter than one hour and those with data missing in the pre- and during-KC periods^6^. During the study period, parents of 66 preterm infants maintained a KC diary. In total, 1407 KC sessions were annotated of which 450 sessions were excluded according to the criteria mentioned above (mostly due to missing ECG data in the pre- and during-KC periods), yielding a database of 957 KC sessions. Characteristics of the 66 infants and their corresponding 957 KC sessions are shown in Table 1. Since the study was observational, the medical ethical committee of the hospital approved the study by providing a waiver in accordance with the Dutch law on medical research with humans (WMO). Informed consent was obtained from all parents filling in the bedside-diaries.

**Table 1:** Patient characteristics at birth and on the days corresponding to Kangaroo care sessions.

Characteristics	Median	IQR
Gestational age (GA) (weeks)	28.8	27–30.4
GA ≤ 28 weeks (n = 27)	26.9	25.6–27.4
GA > 28 weeks (n = 39)	30.3	29–31.1
Birth weight (BW), g	1155	950–1330
ELBW (BW < 1000 g), g (n = 22)	845	740–950
VLBW (1000 g ≤ BW < 1500 g), g (n = 37)	1255	1139–1330
LBW (1500 ≤ BW < 2499 g), g (n = 7)	1650	1621–1739
Length of stay in NICU, day	10.23	5–23.44
Number of KC sessions per infant	14.5	6–32
Duration of KC per session, min	100	81–120
PMA for all KC sessions, week	31	29.6–32.4
Postnatal age for all KC sessions, day	16	8–31

Abbreviations: IQR, interquartile range; ELBW, extremely low birth weight; VLBW, very low birth weight; LBW, low birth weight; PMA, Postmenstrual Age.

### Signal processing for calculating HRV

A peak detection algorithm was used to detect the R-peaks in the ECG recordings^23^ following which four features of HRV, the SDNN, root mean square of successive differences (RMSSD), percentage deceleration (pDec) and standard deviation of the deceleration (SDDec) were calculated for the epochs representative of regulation in the incubator and during KC, as mentioned in the previous section^6^. Similar to previous research, HRV-features were calculated every minute using a moving average window of the past five minutes^6^.

Regarding the physiological implication of the features employed – the SDNN and RMSSD are commonly used features of HRV. The SDNN captures overall HRV, whereas the RMSSD captures short-term or immediate (from one beat to the next beat) variation, and thus these features provide complementary information. In adults, this short-term variation specifically reflects changes driven by the (rapidly acting) parasympathetic nervous system^2^. However, in preterm infants, previous research has shown that SDNN and RMSSD respond in a comparable manner^6–8^. Therefore, owing to its simpler mathermatical construct, we retain SDNN for the primary analysis while relgating the analytical results corresponding to the RMSSD to the appendix. Further, we included in the analysis, two features specifically tailored for the physiology of preterm infants, termed pDec and SDDec. Together, these capture the proportion of heartbeats that were decelerative and the extent of the transient decelerations, reflecting regulatory instability^6^. pDec is defined as the percentage of NN-intervals longer than the average NN-intervals of the past five minutes and reflects the propensity towards decelerations while the SDDec measures the standard deviation of all NN-intervals contributing to pDec and measures the severity of decelerations^6^. In the current study, we, therefore, analyzed the relationship between the SDNN, pDec and SDdec and relevant demographic and maturational factors using statistical models. Results of the analysis corresponding to RMSSD are shown in Online Appendix I. Online Appendix II shows changes in SDNN, pDec, and SDDec in response to KC visualized on a minute-to-minute basis (mean ± SEM) for the entire duration of pre-KC, the first and last 30 minutes of KC, and the entire duration of post-KC.

### Statistical modeling

Linear mixed-effects models (LMM, fixed effects + random effects), an extension of regression models, were used to characterize the relationship between independent variables and the three features of HRV, the SDNN, pDec, and SDDec, i.e., the dependent variables. The LMM approach allows for estimating the individual contribution of each independent variable to the dependent variable and can thus identify how the independent variables relate to and influence the dependent variable.

The fixed effects (FE) correspond to all independent variables and any possible interactions that were coded between them. Independent variables included infant-related demographic variables (GA, PMA, BW, and gender) and the mean value of cardiorespiratory variables (HR and BR) of the period being analyzed (see Fig. 1). For the KC-period, three additional independent variables – the gender of parent performing KC, the duration of KC and pre-KC HRV values, i.e., baseline values of HRV, were also included. Thus, there were 6 and 9 independent variables respectively for the models corresponding to the pre- and during-KC period. Further, interaction terms between GA and PMA (GA × PMA) and between BW and PMA (BW × PMA), for the sub-categories of GA and BW in Table 1, were incorporated into the model because potentially, developmental trajectories after birth may depend on characteristics at birth^24^.

The DVs were the mean values of HRV features (SDNN, pDec, and SDDec) from the representative pre- and during-KC periods respectively, thus giving rise to 6 regression models as each HRV-feature was modeled for two periods. All independent and dependent variables corresponding to these models are characterized (median, IQR) in Table 2. Missing data were imputed by replacing with the mean value of the data set.

**Table 2:** Independent variables and dependent variables for regression models. The ‘Missing’ column indicates the percentage of values that were missing and were imputed.

Independent variables		Data type	Median (IQR)	Missing (%)
**Gender**		Categorical	Male: 45% KCs	0
**Gender of the parent performing KC**		Categorical	Mother: 61% KCs	3.8
**Gestational age (GA), day**		Numerical	197 (188–209)	0
GA ≤ 28 weeks (48% of KCs)		Numerical	188 (177.5–189)	0
GA > 28 weeks (52% of KCs)		Numerical	208 (201–213)	0
**Postmenstrual age, day**		Numerical	218 (209–226)	0
**Birth weight (BW), g**		Numerical	1030 (830–1275)	0
ELBW (<1000 g), (49% of KCs)		Numerical	830 (680–935)	0
VLBW (1000–1500 g), (44% of KCs)		Numerical	1275 (1120–1330)	0
LBW (1500–2499 g), (7% of KCs)		Numerical	1765 (1640–1860)	0
**Duration of KC, min**		Numerical	100 (81–120)	0
**Heart rate (bpm)**	Pre-KC	Numerical	160.4 (153.4–167.4)	0.1
	During-KC	Numerical	159.5 (153.3–165.3)	0.3
**Breathing rate (brpm)**	Pre-KC	Numerical	52.35 (45.6–60.6)	1.6
	During-KC	Numerical	49.5 (43.1–57.1)	1.5
**Dependent variables**		**Data type**	**Median (IQR)**	**Missing (%)**
**SDNN (ms)**	Pre-KC	Numerical	24.33 (24.05–24.70)	0
	During KC	Numerical	21 (20.7–21.7)	0
**pDec (%)**	Pre-KC	Numerical	46.47 (46.36–46.74)	0
	During KC	Numerical	45.80 (45.25–46.72)	0
**SDDec (ms)**	Pre-KC	Numerical	24.38 (23.96–25.05)	0
	During KC	Numerical	22.14 (21.61–22.97)	0

Abbreviations: KC, Kangaroo care; KCs, Kangaroo care sessions; IQR, interquartile range; GA, gestational age; bpm, beats per minute; brpm, breaths per minute; BW, birth weight; SDNN, standard deviation of normal to normal intervals; pDec, percentage of decelerations; SDDec, standard deviation of decelerations.

Finally, the hierarchical structure of the data consists of infants and KC sessions within infants. This is incorporated as a random effect (RE) in an LMM in the form of a random intercept for each individual infant. In effect, the RE-term corresponding to each infant was intended to quantify that aspect of inter-infant variability that remains uncaptured in FEs, as well as to strengthen the assumption of independence between KC sessions, which nevertheless were a day or further apart, typically. The ratio of the variance of the random intercept to the total variance, also known as the intra-class correlation, was calculated to explain the overall variance in the HRV that could be explained by infant-level clustering.

### Model development and diagnostics

All independent variables under consideration were included in the LMM since, based on calculating correlations between variables, there was little evidence of multicollinearity. In general, correlations were low (typically <0.3), except for a moderate correlation between GA and BW (0.68) which was expected. The purpose of the model was to unravel the influence of these two effects.

Concerning DVs, the LMM is more likely to be appropriate for DVs that are roughly normally distributed. Therefore, the SDNN and SDDec, which happened to have a skewed distribution, were log-transformed before modeling. Post model development, several regression diagnostics procedures were used to assess the validity of the models developed and to ascertain whether the statistical assumptions for regression modeling were satisfactorily met. These checks included generating the following:

1. Normal probability plots of the residuals of the regression model to assess whether the residuals were normally distributed.
2. Plots of fitted values versus the residuals to identify heteroscedasticity.
3. Plots of independent variables and residuals to determine whether there were any trends in the data suggesting that the independent variables needed prior transformation, for instance, squaring or log-transformation.
4. Plots of residuals versus leverage, with overlaid Cook’s distance contour plots to identify and characterize the effect of any outliers. Leverage measures how far an observation is from the mean value of the remaining observations and in effect measures the unusualness of the observation. Cooks-distance is a measure of the influence of an observation in changing the slope of the regression line.
5. The histogram of the RE to identify whether the random intercept was roughly normally distributed and that no individual infant exhibits patterns distinctly different from the rest.

### Model interpretation and statistical testing

The F-test was used to compare the results of the regression model incorporating only FEs to an intercept-only model while the log-likelihood ratio test was used to compare the effect of adding REs to the FE-only model. The goodness-of-fit of all models was quantified by the coefficient of determination, i.e., the adjusted R^2^.

Estimates of individual regression coefficients characterized the effect of each independent variable on the dependent variable, thus allowing for conclusions regarding independent variables that added information to one another, where the effect of each independent variable can be interpreted as the effect of that variable while holding all other independent variables constant. For the interacting independent variables, if the interaction was statistically insignificant, then only the *main effects (*average effect across all levels of the interacting variable) were shown. If the interaction was statistically significant, main effects and interacting effects were discussed individually. Appropriate contrast matrices were used to identify the p-values corresponding to these main effects. Statistical significance was defined as a p-value < 0.05.

Since the SDNN and SDDec were log-transformed, in effect, the LMM modeled DVs which had a non-linear relationship with the independent variables. Therefore, for ease of interpretation, instead of providing the regression table we plot the effect of all independent variables (with 95% confidence intervals, CI) against the suitably transformed dependent variable, along with the corresponding p-values of the regression coefficient. For these plots, the independent variable was varied between the 5^th^ to 95^th^ percentile ranges of values, as estimated from the dataset, while all other independent variables were held constant at their corresponding median levels. All data were analyzed using Matlab R2017a Matlab (MathWorks).

## Results

We analyzed 957 KC sessions in 66 preterm infants, 32 male, and 34 females. Their median GA was 28.8 (27–30.4) weeks while their median PMA on days corresponding to KC sessions was 31 (29.6–32.4) weeks. For other demographic data, we refer to Table 1.

All six regression models were statistically significant (p-value < 0.001) with FEs alone; however, adding REs improved the fit of all models to the data (p-value < 0.001). Table 3 provides the R^2^ values for models with just the FEs alone as well as for the models using both FEs and REs. Since R^2^ increases upon adding REs, incorporating infant-to-infant variability improves the goodness of fit of the regression models. Further, the intra-class correlation, as shown in Table 3, gives an indication of the fraction of the total variation in HRV-features that is explained by infant-level clustering.

**Table 3:** The goodness of fit, as measured by the R^2^, for all six regression models with FE alone as well as the R^2^ and the intra-class correlation (ICC) for the model with both FE and RE.

Period	IV	R^2^ with FE	R^2^ with FE and RE	ICC
Pre-KC	SDNN	0.06	0.18	0.15
	pDec	0.12	0.26	0.19
	SDDec	0.04	0.09	0.07
During-KC	SDNN	0.13	0.23	0.12
	pDec	0.29	0.42	0.19
	SDDec	0.12	0.17	0.08

**Figure 2:**
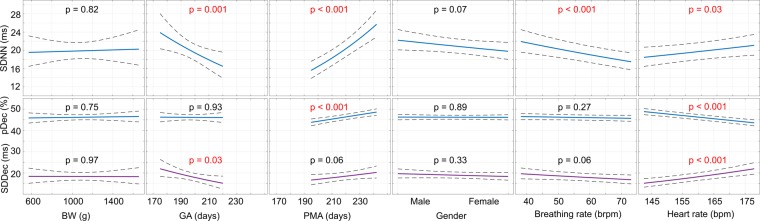
Regression plots showing how the independent variables affect the SDNN (1^st^ row), and pDec and SDDec (2^nd^ row) when the infant was in the incubator. Each column reflects an independent variable, as annotated on the x-axes. The y-axes show the values of the HRV features after suitable transformation. Statistically significant (p-value < 0.05) variables are in red.

**Figure 3:**
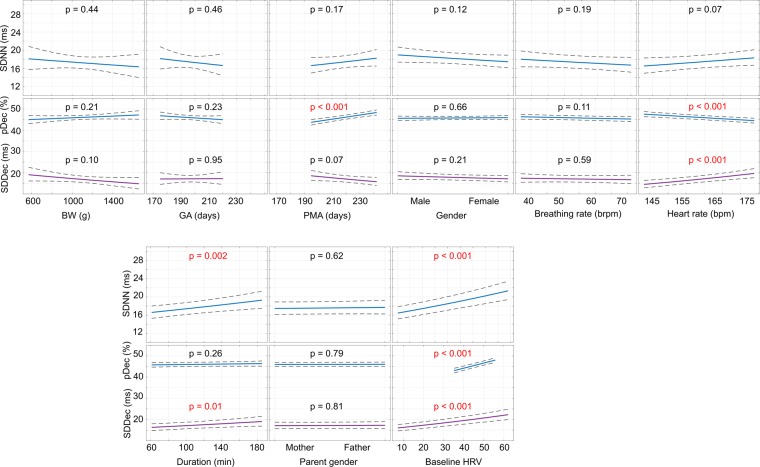
Regression plots showing how the independent variables affect the SDNN (1^st^ and 3^rd^ row), and pDec and SDDec (2^nd^ and 4^th^ row) during KC. Columns reflect different independent variables, as annotated on the x-axes. The y-axes show the values of the HRV features after suitable transformation. Statistically significant (p-value < 0.05) variables are in red. The first two rows show those independent variables which were also incorporated into the HRV-model while the infant was in the incubator while the last two rows show additional Kangaroo care-related variables.

Figures 2 and 3 show the plots corresponding to how each independent variable affects the features of HRV, along with whether the relationship was statistically significant. Figure 2 corresponds to the model of routinely exhibited HRV, i.e., HRV modeled during a stable period in the incubator (pre-KC). Figure 3, on the other hand, corresponds to how infants responded to KC with the inclusion of three additional independent variables to the pre-KC model – the gender of parent performing KC, the duration of KC, as well as baseline levels of HRV obtained from the pre-KC period.

Figure 2 shows that, as measured by the SDNN, the GA and PMA strongly influence overall HRV. The SDNN reduces with increasing GA, while it increases with increasing PMA. Analyzing the contribution of transient decelerations (pDec and SDDec) to overall HRV (SDNN) shows that the reduction in SDNN with increasing intra-uterine development (i.e., increasing GA) is correlated with a reduction in the severity of decelerations (SDDec) while the ratio of decelerations to accelerations (pDec) remains unaffected and remains, on average, below 50%, throughout. In this regard, it appears that the GA and not the BW influences the developmental trajectory of the autonomic nervous system. With increasing post-uterine maturation, i.e., PMA, pDec starts increasing toward 50% while the SDDec remains largely uninfluenced (p-value > 0.05). As a result, overall HRV reflected by the SDNN increases with increasing PMA. BR and HR also influence SDNN but in comparison to GA and PMA, to a smaller extent. A higher HR is associated with a higher SDNN due to a propensity towards fewer but more severe decelerations, while a higher BR is associated with lower SDNN. Gender and BW have no apparent effect on HRV.

During Kangaroo care (Fig. 3), the overall HRV as reflected by the SDNN is principally affected by baseline levels of SDNN measured in the incubator. Increasing duration of KC increases SDNN while GA and PMA have no additional effect. Regarding pDec and SDDec, increasing the PMA results in pDec values that are closer to 50%. Similar to in the incubator, a higher HR reduced pDec while increasing SDDec. BR, BW, infant gender and the gender of the parent performing KC did not influence HRV during KC.

Further, no interactions were statistically significant except the interaction between GA and PMA in the model of pDec as a DV for the pre-KC period. This interaction plot is shown in Online Appendix III; briefly, with increasing PMA, pDec increased at a faster rate in infants born less than 28 weeks GA versus those born after 28 weeks of gestation. Concerning model diagnostics, the tests were deemed satisfactory; exemplary examples of the diagnostic plots are shown in Online Appendix IV.

## Discussion

In this study, we quantified the influence of several demographic and cardiorespiratory factors on the autonomic regulatory mechanisms of preterm infants, as measured by HRV, during periods in the incubator versus during periods of KC. To model how features of HRV are affected by these factors, we used a statistical approach and specifically tailored features of HRV to capture the unique physiology of preterm infants.

Regression analysis demonstrated that GA and PMA had differing effects on the physiological mechanisms affecting HRV. Increasing PMA increased SDNN while increasing GA reduced SDNN while holding all other independent variables constant. Potentially these differences can be attributed to distinct trajectories of intra- and extrauterine development. Gender and BW did not affect values of HRV, as found in a previous study as well^9^.

The reduction in SDNN with increasing GA was associated with a reduction in the extent of decelerations (SDDec) while the percentage of decelerations (pDec) remained remarkably constant. Increasing PMA, on the other hand, increased SDNN and this was driven primarily by an increase in the percentage of decelerations. In fact, with increasing PMA, the pDec increased toward 50% while the SDDec remained largely unchanged. The fact that with an increasing PMA, the pDec tends to increase towards 50% might be a reflection of increasing maturity since the ratio of decelerations to accelerations that is close to unity has been described as *healthy* in preterm infants^12^. Similarly, increasing values of SDNN have been associated with increasing maturation in previous studies, but since those studies do not stratify findings by GA and PMA, our analysis adds a novel perspective^9,25,26^.

Increasing values of SDNN due to increasing PMA might be attributable to increasing myelination of the parasympathetic nervous system that starts around week 30–31 weeks PMA^8,27^. Greater myelination results in faster-acting and more stable regulation with an enhanced capacity to respond to instantaneous environmental changes^28^. Along the lines of Porges’ Polyvagal theory, we speculate that the high SDNN owing to severe decelerations in infants of low GA reflects immature autonomic regulation due to the unmyelinated branches of the parasympathetic nervous system, as described previously^6,29^. This argument is supported by the fact that pDec remains unaffected by changes in GA, but increases toward 50% with increasing PMA. Remarkably, the effect of prolonged intra-uterine development (i.e., increasing GA) on pDec and SDDec while the infant is in the incubator, is similar to the effect that KC exerts on HRV (pDec relatively unaffected, SDDec reduced). In other words, in preterm infants, KC appears to temporarily improve autonomic regulation as seen with increasing developmental maturity.

Concerning the factors affecting HRV during KC, the largest effect was that of baseline values of HRV, i.e., from the pre-KC period. However, despite adjusting for baseline HRV which already captures the contribution of PMA, during KC, HRV was still affected by the PMA. This finding affirms previous results suggesting that the response to KC is affected by the maturational state of the infant^7,8^. While in the incubator, with increasing PMA, pDec approaches 50% but by performing KC there appears to be further PMA-dependent catalysis that facilitates pDec towards 50%.

During KC, other factors, barring the duration of KC, did not affect overall HRV. Increasing duration of KC was associated with increasing HRV, but this finding was artefactual. Based on the clinical workflow in our unit, during long KC sessions, we know that there is an increased likelihood of nursing care taking place (typically scheduled for once every two hours), which includes feeding and handling – well-known stressors that tend to increase HRV^21^. Upon performing the regression analysis while excluding KC sessions that were longer than 3 hours, the effect of KC-duration on HRV disappeared entirely indicating that in the original analysis, KC-duration did not affect HRV.

HR, on the other hand, did influence HRV, both during periods in the incubator as well as during KC. A higher HR skewed the ratio of decelerations and accelerations; decelerations became fewer but more severe. During periods in the incubator, this increased SDNN. A high HR is likely an expression of a high sympathetic tone – in adults such a state tends to reduce HRV by adopting a steady and unvarying HR^1^. In preterm infants, a high HR appears to increase the propensity towards extensive decelerations manifesting itself as a high SDNN, not unlike the increased SDNN seen in fetuses in the third trimester of pregnancy during periods of increased sympathetic tone^30^.

As opposed to the HR, BR had a less evident effect on HRV – higher BR reduced SDNN and SDDec, while pDec remained unchanged. Since pDec remained unchanged, we speculate that the effect of BR on HRV is not primarily due to changes in autonomic regulation, but rather owing to a reduction in the mechanical effect of breathing on HRV. Faster and thus shallower breathing may modulate HRV to a lesser extent owing to reduced respiratory period and depth^31,32^.

The strengths of this study include using a large dataset obtained from a sizable patient population for the analysis. Further, we decomposed the effect of several key demographic and cardiorespiratory factors to identify factors that independently affect HRV and thus autonomic regulation. Limitations of the study include the fact that not all possible factors that may affect HRV, for instance, sleep-states and position of infants were accounted for. Further, only a limited amount of data corresponding to extremely preterm infants (24–28 weeks GA and PMA) was present, impairing specific analysis for this important group. Since regression outcomes cannot be extrapolated, this is a limitation that should be addressed in future research. Additionally, since the study was of an observational nature, we did not restrict nursing activity during long KC sessions. Finally, while we excluded KC sessions during which infants were mechanically ventilated, in the analysis, we did not adjust for alternative methods of respiratory support for spontaneously breathing infants.

In conclusion, autonomic regulation in preterm infants is distinctly affected by intra- versus extra uterine maturation. With increasing GA, the propensity towards few but severe pathophysiological HR decelerations appears to decrease which in turn tends to reduce overall variability. With increasing PMA, on the other hand, the ratio of decelerations and accelerations approaches unity which contributes to an increase in overall HRV. By helping unravel the factors modulating the autonomic regulatory mechanisms in preterm infants, we foresee the results of our study in contributing towards individualizing care, tracking the maturational trajectories of preterm infants and in predicting disease.

## Supplementary information

Statistical Modeling of Heart Rate Variability to Unravel the Factors Affecting Autonomic Regulation in Preterm Infants
