# Supplementary material for: Statistical Modeling of Heart Rate Variability to Unravel the Factors Affecting Autonomic Regulation in Preterm Infants
Source: Sci Rep. 2019 May 22;9:7691. doi: 10.1038/s41598-019-44209-z (PMC6531452; doi:10.1038/s41598-019-44209-z)
Supplement: Supplementary file 1 — Statistical Modeling of Heart Rate Variability to Unravel the Factors Affecting Autonomic Regulation in Preterm Infants [file 41598_2019_44209_MOESM1_ESM.docx]

Online Appendices

**Statistical Modeling of Heart Rate Variability to Unravel the Factors Affecting Autonomic Regulation in Preterm Infants**

Rohan Joshi^1,2,3^, Deedee Kommers*^4,5^, Chengcheng Guo^6^, Jan-Willem Bikker^7^, Loe Feijs^1^, Carola van Pul^2,5^, Peter Andriessen^4^

^1^ Department of Industrial Design, Eindhoven University of Technology, The Netherlands.
^2^ Department of Clinical Physics, Máxima Medical Centre Veldhoven, The Netherlands.

^3^ Department of Family Care Solutions, Philips Research, Eindhoven, The Netherlands.

^4^ Department of Neonatology, Máxima Medical Centre, Veldhoven, The Netherlands.
^5^ Department of Applied Physics, Eindhoven University of Technology, The Netherlands.

^6^ Department of Electrical Engineering, Eindhoven University of Technology, The Netherlands

^7^ Consultants in Quantitative Methods, CQM BV, Eindhoven, Netherlands

Appendix I – Regression plots showing how the independent variables affect the RMSSD while the infant was in the incubator.


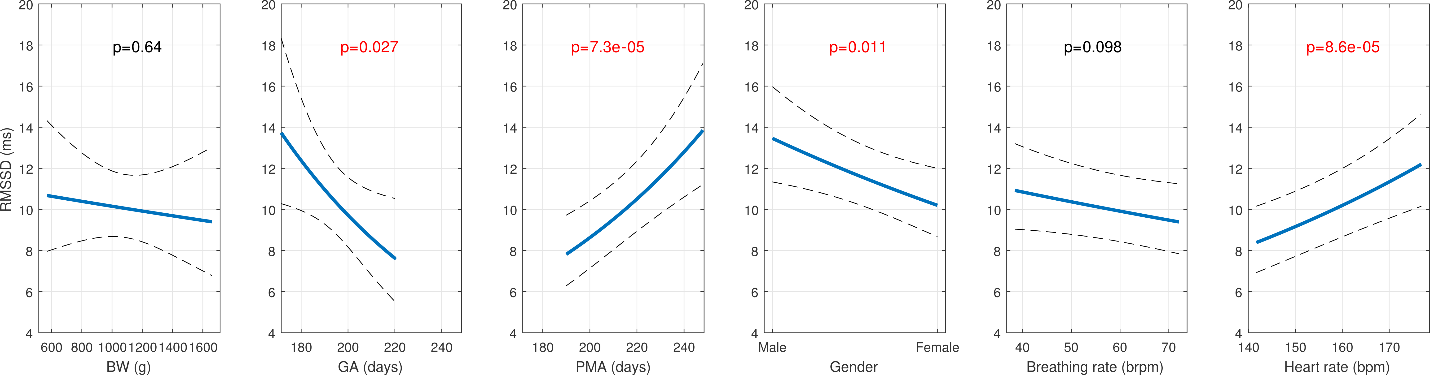


Each column reflects an independent variable, as annotated on the x-axes. The y-axes show the value of the RMSSD after suitable transformation. Statistically significant (p-value < 0.05) variables are in red.

Appendix II – Dynamic changes in SDNN, pDec and SDDec in response to KC


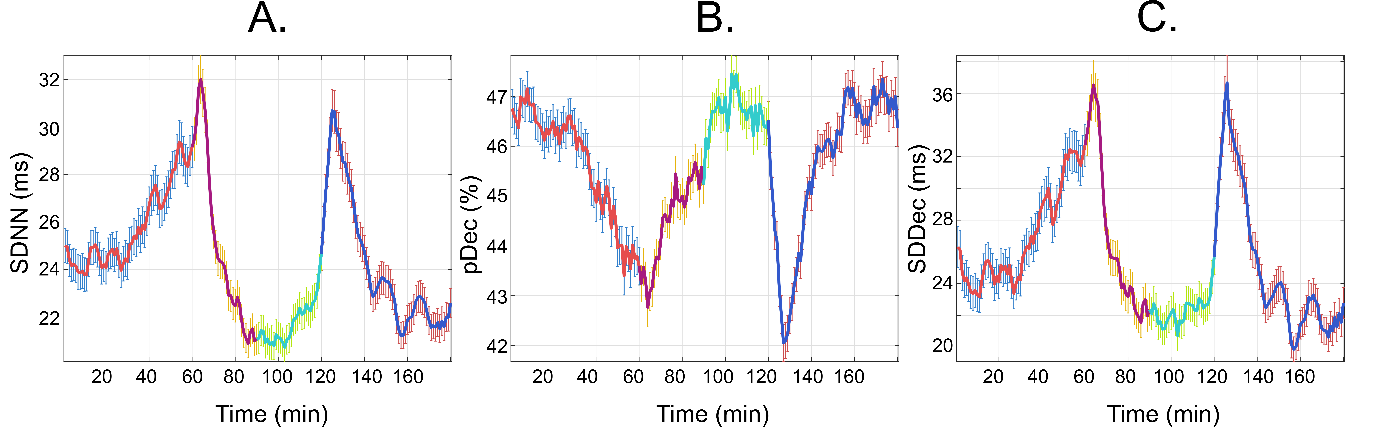


Time series of three features of heart rate variability (HRV) in the incubator before Kangaroo care (KC) starts (1-60th minute), during the first and last 30 minutes of KC (60-120th minute), and after KC (120-180th minute). The mean±SEM values of the three features – the standard deviation of all NN-intervals (SDNN, Figure A), the percentage of decelerations (pDec, Figure B), and the standard deviation of decelerations (SDDec, Figure C) – are shown for all 957 KC sessions.

Appendix III – Interaction plots


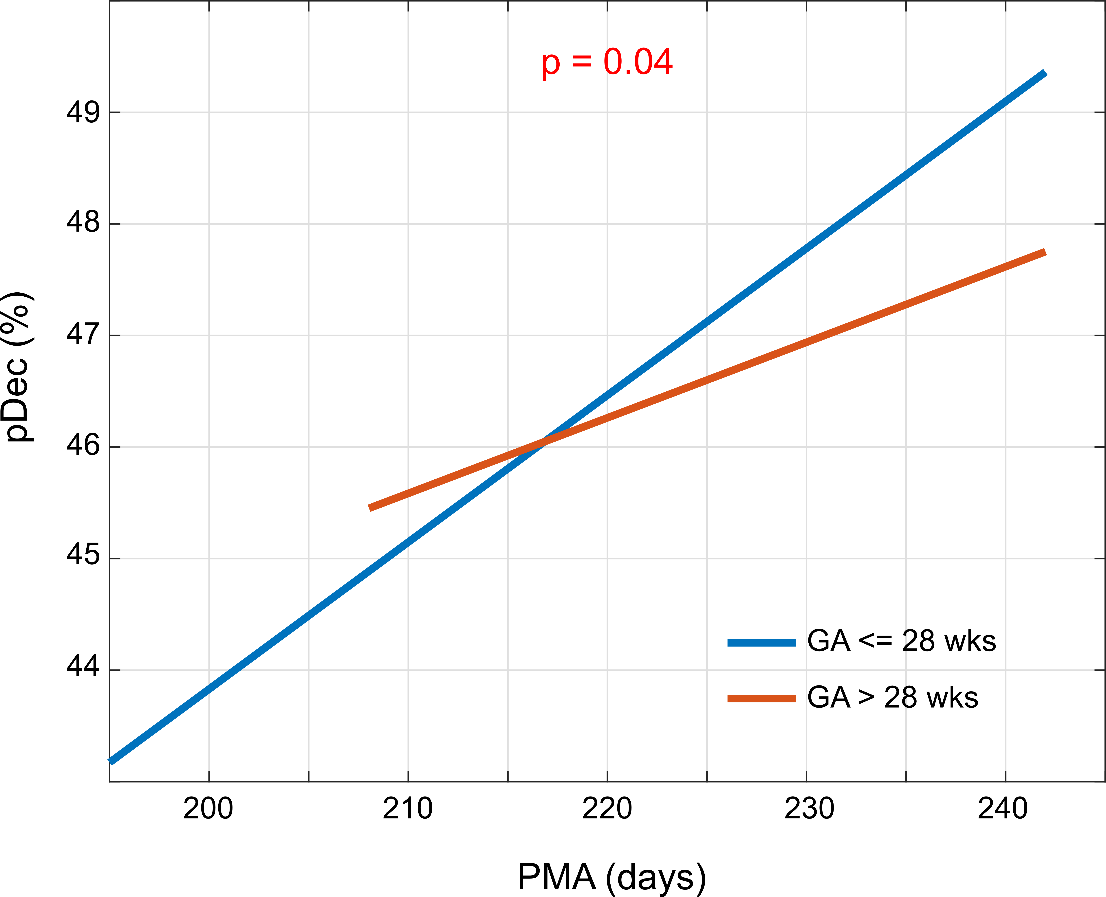


There was a statistically significant interaction between GA and PMA, with the rate of increase of pDec, with respect to PMA, steeper in infants born below 28 weeks of gestation that those born after 28 weeks of gestation. Nevertheless, for both infants born before and after 28 weeks of gestation, pDec increased with increasing PMA and the differences in pDec for different PMA between the two infant groups was small. Therefore we only calculate the main effect of increasing PMA on pDec, irrespective of GA.

Appendix IV – Model diagnostics

In the following figures, we show exemplary examples of model diagnostics corresponding to the model of SDDec during the period in the incubator.

1. Normal probability plot


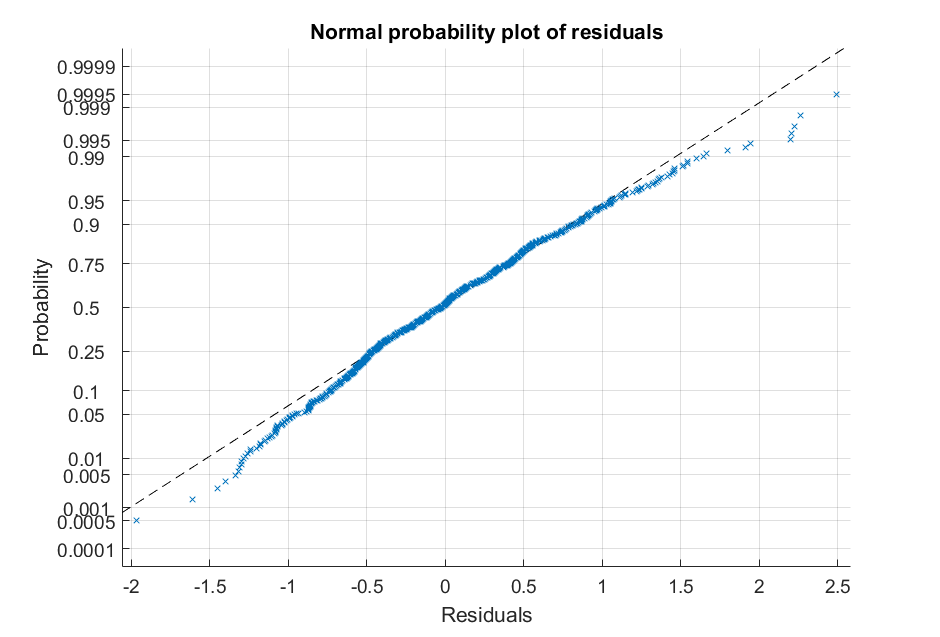


While there are a few outliers in the tails, the largely diagonal distribution of the residuals indicates that, by and large, the distribution of residuals was normally distributed.

1. Fitted values versus residuals


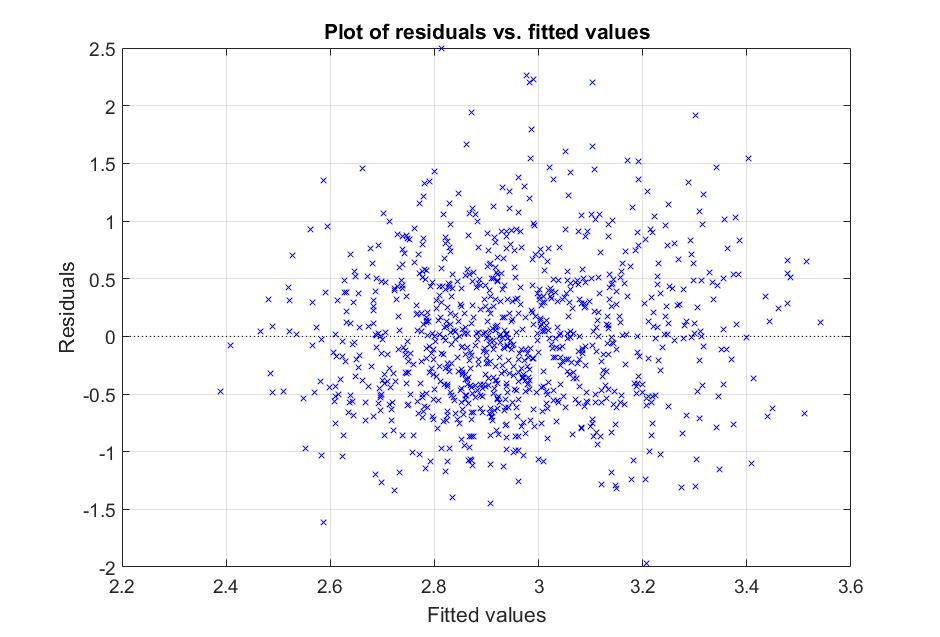


The residuals appear to be randomly distributed around the fitted values and have no predictive value, suggesting that the model is sufficiently homoscedastic.

1. The plot of birth weight, an independent variable, versus residuals.


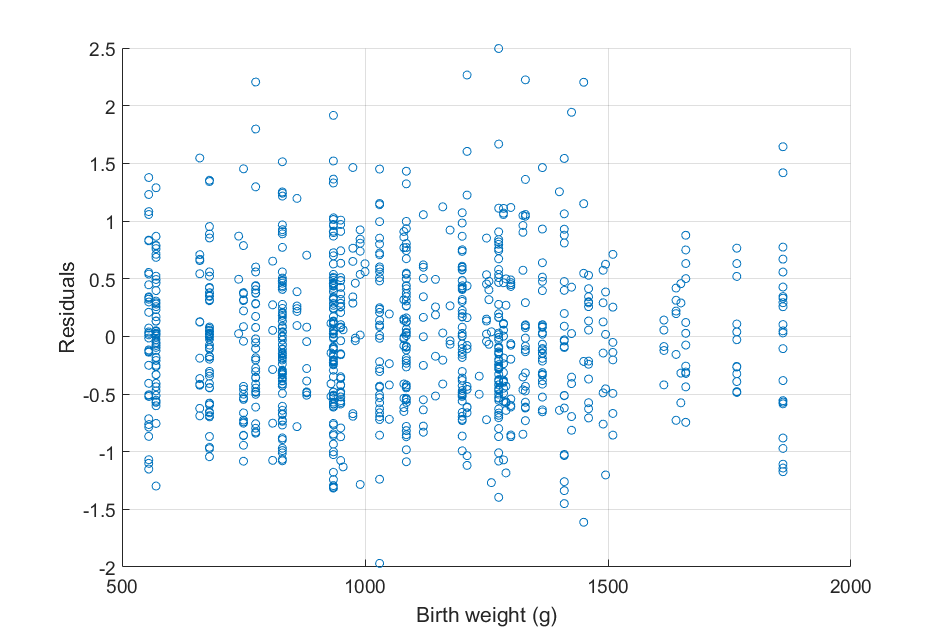


1. A plot of residuals versus leverage with overlaid Cook’s distance.


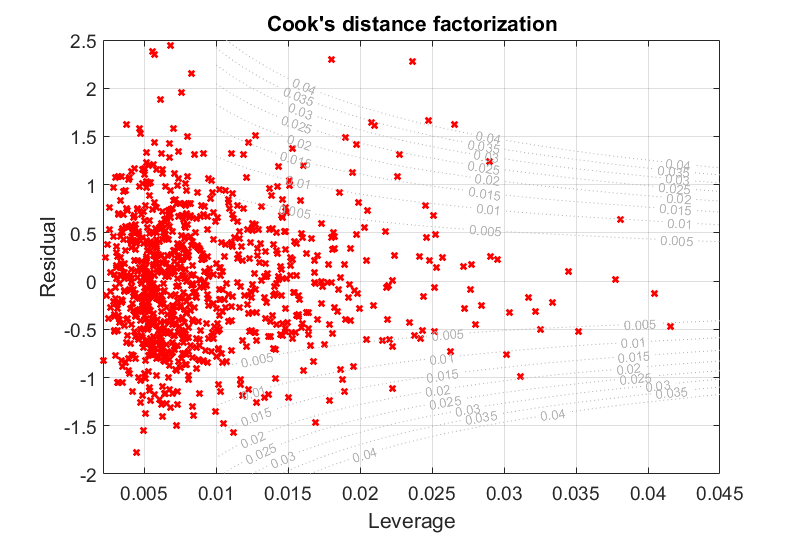


Concerning Cooks-d, there are no obvious outliers or substantially large values. Also, the absolute values of Cook-d are considerably smaller than for instance, the often used rule of thumb of 1. Concerning leverage, there are practically no points of high leverage that have a large Cooks-d, allowing us to discern that there are no notable outliers in the data.

1. Histogram of random intercepts


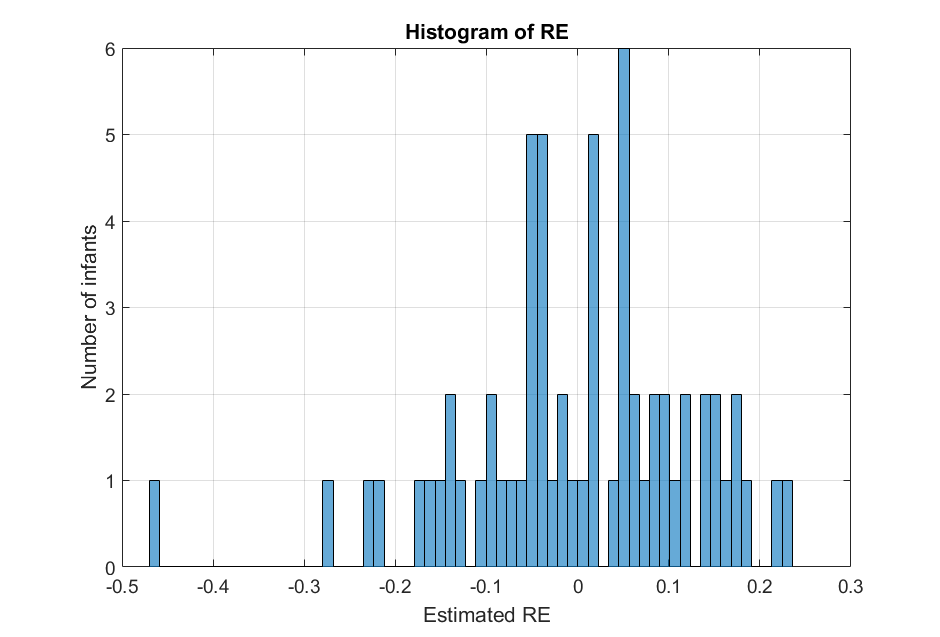


Roughly, the estimates of the random intercepts are normally distributed.
